# Anti-hemorrhagic Activity of Four Brazilian Vegetable Species Against *Bothrops jararaca* Venom

**DOI:** 10.3390/molecules14031072

**Published:** 2009-03-09

**Authors:** Catarine Massucato Nishijima, Clenilson Martins Rodrigues, Marcelo Aparecido Silva, Mônica Lopes-Ferreira, Wagner Vilegas, Clélia Akiko Hiruma-Lima

**Affiliations:** 1 Department of Physiology, São Paulo State University, UNESP/Botucatu-SP, Brazil; 2 Department of Organic Chemistry, São Paulo State University, UNESP/Araraquara-SP, Brazil; 3 Center of Applied Toxinology. Butantan Institute, São Paulo-SP, Brazil

**Keywords:** Flavonoids, Antiophidic plants, Anti-hemorrhagic, *Bothrops jararaca*

## Abstract

Around 20,000 snakebites are reported annually in Brazil and 90% of them are inflicted by species of the genus *Bothrops*. Intravenous administration of antibothropic antivenom neutralizes the systemic actions, but it is of little effect on the reversal of local symptoms and often induces adverse reactions, a context that drives the search for complementary treatments for snakebite accidents. Vegetable extracts with a range of antiophidian activities constitute an excellent alternative. In this study, we investigated the anti-hemorrhagic effects of *Mouriri pusa* Gardn. (Melastomataceae), *Byrsonima crassa* Niedenzu (Malpighiaceae), *Davilla elliptica* St. Hill. (Dilleniaceae) and *Strychnos pseudoquina* St. Hil. (Loganiaceae) against *Bothrops jararaca* venom. The methanolic extracts from *M. pusa* (leaves), *B. crassa* (leaves) and *D. elliptica* (leaves) showed total neutralization capacity against local hemorrhages. The amenthoflavone and quercetin fractions from *B. crassa* and the flavonoids fractions (quercetin and myricetin) from *M. pusa* and *D. elliptica* also showed total neutralization capacity. We conclude that flavonoids derived from myricetin, quercetin and amenthoflavone play an important role in the anti-hemorrhagic potential of these Brazilian vegetables species against *B. jararaca* venom.

## Introduction

Annually, *Bothrops* accounts for more than 90% of snakebite accidents in Brazil [1], with *Bothrops jararaca* being responsible for a major portion of these ophidic accidents [2]. Venom from *Bothrops jararaca* induces local reactions such as edema, pain, hemorrhage and necrosis, besides systemic reactions, mainly represented by blood coagulation disturbances. Hemorrhages represent one of the most conspicuous toxic activities in bothropic envenomation. Hemorrhages are principally caused by Zn^2+^-dependent metalloproteinase (MMP) enzymes, which are responsible for degrading proteins of the extracellular matrix. The MMPs also have a cytotoxic effect on endothelial cells and act on components of the hemostatic system [3]. Some metalloproteinases induce hemorrhage by directly affecting mostly capillary blood vessels by clearing in a highly selective fashion key peptide bonds of basement membrane components, thus affecting the interaction between basement membrane and endothelial cells [4]. The antibothropic serum is the specific treatment for envenomation by *Bothrops* snakes. This therapeutic antivenom is produced by the immunization of horses with venoms from a limited number of *Bothrops* species. Through serum therapy, the neutralization of the systemic toxic effects is usually achieved but the neutralization of local tissue damage usually does not occur. Thus, the search for novel venom inhibitors capable of complementing serum therapy appears promising. Muniz *et al*. [5] reported that Brazilian *Bothrops* polyspecific antivenom is not very efficient in neutralizing the effects of venoms of some *Bothrops* species from the Amazonian rain forest, a context that drives the search for complementary treatments to minimize venom injuries [6].

Plant extracts contain a large diversity of chemical compounds displaying several pharmacological activities. Wagner [7] described an herbal drug with superior efficacy and lesser side effects in comparison with single isolated constituents, mainly in multifactorial pathophysiology, which is elucidated by the synergistic interactions in phytomedicine that explain the efficacy of apparently low doses of active constituents of herbal products [8]. Some authors reported the use of medicinal plants as an anti-venom agent by applying these species to snakebites in order to neutralize the effects of the venom [9]. Some authors demonstrated that extracts rich in proanthocyanidins, triterpenoid saponins, poly-saccharides and coumarins are powerful inhibitors of bothropic venom metalloproteinases [10,11,12]. Flavonoids have been deemed responsible for anti-inflammatory, antiulcerogenic, healing, antihypertensive and many other activities. Some of these compounds exert inhibition of phospholipase A2, an important components of snake venoms [10]. The present study evaluated the potential anti-hemorrhagic effect of vegetal extracts and flavonoids fractions obtained from *Mouriri pusa*, *Byrsonima crassa*, *Davilla elliptica* and *Strychnos pseudoquina.*

## Results and Discussion

Medicinal plants play a key role in world health, as they are sources of many pharmacologically active compounds such as flavonoids. The use of plant extracts as antidotes for animal venoms has become an important research objective that seeks a complementary therapy for antisera. 

Flavonoids are a group of about 4,000 naturally occurring compounds that are ubiquitous in all vascular plants [13]. Castro *et al.* [14] identified secondary metabolites in extracts of plants that totally neutralized the hemorrhagic effect of venom from *B. asper*. These same authors suggested that flavonoid-rich extracts could be chelators towards ions such as zinc, a necessary element for the enzymatic activity of metalloproteinases. Furthermore, flavonoids are known to be involved in vascular protection, by decreasing the vascular permeability and inhibitory activity of several enzymes. Many synthetic inhibitors of metalloproteinases, such as EDTA, act through mechanisms based on metal chelation, which is necessary for catalysis [15,16].

The hemorrhagic activity of *Bothrops* venoms alters the homeostasis of the blood vessel walls and causes their rupture [17]. In our research, the dose of *B. jararaca* venom that induced a mean hemorrhage halo of 10 mm diameter in 2 hours was 8.1 μg, the venom dose that we utilized in the present study in all experiments with the intent to standardize the hemorrhage halo. 

*Byrsonima crassa* is a medicinal plant used in traditional medicine to treat gastrointestinal disorders and snakebite. Phytochemical investigation [18] revealed the occurrence of amentoflavone, quercetin 3-*O*-β-D-galactopyranoside and quercetin 3-*O*-α–L-arabinopyranoside from the extract of *Byrsonima crassa* leaves. We can observe the importance of this amentoflavone in anti-hemorrhagic activity of *B. crassa* in Table 1. Both methanolic extract and rich flavonoids fraction showed total neutralization capacity (100%) against *Bothrops jararaca* hemorrhage (Table 1). Mors *et al*. [7] already reported that quercetin and several of its glycosides are the flavonoids most often encountered in plants with anti-snake-venom properties. 

The capacity to totally neutralize venom was also observed when venom was challenged with the methanolic and flavonoid extracts from *Mouriri pusa* and *Davilla elliptica* (Table 1). These two species, belonging to different botanical families, also present rich flavonoid fractions with quercetin and several of its glycosides. The flavonoid fraction from *Mouriri pusa* consists mainly of kaempferol, myricetin, and flavonoids derived from quercetin [19] while the flavonoids-rich fraction from *D. elliptica* consists mainly of flavonoids derived from myricetin and quercetin [20]. Mors *et al*. [7] already described some species with kaempferol with anti-snake venom properties. Species such as *Phyllanthus niruri* and *P. urinaria* contain quercetin and its rutin glycosides that have important anti-snake venom properties [7]. The only species without quercetin in its composition was *Strychnos pseudoquina,* a medicinal plant belonging to the Loganiaceae family. The methanolic extract and both fractions (flavonoids and alkaloids) showed partial neutralization potential against hemorrhagic effect (Table 1). This species has a distinct chemical composition when compared to the all other species evaluated in this study, as it has methoxylated flavonoids derived such as isorhametine, strychnobiflavone, quinol, diaboline and 11-methoxydiabolin [21, 22, 23]. Probably these methoxylated flavonoids from *S. pseudoquina* were not able to chelate ion metalloproteinase metal ions with their predominantly 3- and 5-hydroxyl as well as cathecol groups.

**Table 1:** Anti-hemorrhagic effect of methanolic, rich quercetin fractions from and rich amentoflavone fractions from *Byrsonima crassa*, methanolic extract and rich flavonoids fraction from *Mouriri pusa*, methanolic extract and rich flavonoids fraction from *Davilla elliptica*, methanolic, rich alkaloids fraction and rich flavonoids fraction from *Strychnos pseudoquina* against lesions produced by intradermal injection of *B. jararaca* venom.

Treatment (intradermal)		N	Hemorrhagic diameter (mm)	Hemorrhagic area (mm^2^)	Inhibition (%)
Venom	-	6	9.79 ± 0.42	54.95 ± 3.04	-
Venom^a^ +	Methanolic extract from *B.crassa*	6	0**	0**	100
Venom^a^ +	Amentoflavone-rich fraction from *B. crassa*	6	0**	0**	100
Venom^b^	-	7	10.23 ± 0.28	51.69 ± 1.28	-
Venom^b^ +	Methanolic extract from *M. pusa*	7	0 **	0 **	100
Venom^c^	-	7	10.04 ± 0.16	52.72 ± 1.43	-
Venom^c^ +	Flavonoid-rich fraction from *M. pusa*	7	0**	0**	100
Venom^c^ +	Quercetin-rich fractions from *B. crassa*	7	0**	0**	100
Venom^d^	-	6	11.21 ± 0.47	50.74 ± 2.80	-
Venom^d^ +	Methanolic extract From *D. elliptica*	6	0**	0**	100
Venom^e^	-	7	8.77 ± 0.32	46.06 ± 3.53	-
Venom^e^ +	Flavonoid-rich fraction From *D. elliptica*	7	0**	0**	100
Venom^f^	-	7	10.97 ± 0.78	54.30 ± 1.69	-
Venom^f^ +	methanolic extract from *S. pseudoquina*	6	8.05 ± 0.32***	42.95 ± 3.17**	21-27
Venom^f^ +	Flavonoid-rich fraction from *S. pseudoquina*	6	8.18 ± 0.61***	44.95 ± 3.73*	17-25
Venom^f^ +	Alkaloid-rich fraction from *S. pseudoquina*	6	7.87 ± 0.31***	43.95 ± 2.27*	19-28

^a^ANOVA F_(5;30) _= 549.07 for HD (hemorrhagic diameter); F_(5;30) _= 327.76 for HA (hemorrhagic area). ^b^ANOVA F_(5;36) _= 1303.1 for HD; F_(5;36) _=1642.3 for HA. ^c^ANOVA F_(5;36) _= 3745.9 for HD; F_(5;36) _= 364.2 for HA. ^d ^ANOVA F_(5;30) _= 565.27 for HD; F_(5;30) _= 4550.1 for HA. ^e^ANOVA F_(3,24) _= 737.59 for HD; F_(3,24)_=170.40 for HA. ^f^ANOVA F_(7;43) _= 127.96 for HD; F_(7;43) _= 135.19 for HA. Dunnett’s Test *p<0.05, ** p<0.01, *** p<0.001. The values represent mean ± S.E.M.

## Conclusions

Our results suggest that kaempferol, quercetin and glycosides (quercetin 3-*O*-β-D-galactopyranoside and quercetin 3-*O*-α–L-arabinopyranoside), amenthoflavone and myricetin are the main flavonoids present in *Davilla elliptica, Mouriri pusa* and *Byrsonima crassa* that have total venom neutralization capacity against hemorrhagic activity of *Bothrops jararaca.*

## Experimental

### Plant Species

These species employed in this study were collected in Porto Nacional, Tocantins, Brazil. The botanical identification was performed by Dr. Eduardo R. dos Santos and Dr. Solange F. Lolis from the Federal University of Tocantins. A voucher specimen of each species was deposited at the Herbarium HTINS, Federal University of Tocantins as numbers 3291 (*S. pseudoquina* St. Hil., 4549 (*M. pusa* Gardn***.*)**, 3377 (*B. crassa* Nied.) and 4583 (*D. elliptica*). The extracts were prepared by maceration at room temperature using two solvent series (1 week for each solvent): first with chloroform (CHCl_3_) or dichloromethane (DCM) to remove lypophilic compounds, and after with methanol (MeOH). The solvents from each extraction procedure were combined and evaporated at 45ºC under vacuum to yield two extracts, designated CHCl_3_ or DCM extract and MeOH extract. From the air-dried and powdered of leaves of *Strychnos pseudoquina* (300 g) were obtained the DCM extract (13.9 g) and MeOH extract (17.7 g). From leaves of *Mouriri pusa* (1 kg) were obtained the DCM extract (35.0 g) and MeOH extract (100.0 g). From the leaves of *B. crassa* (2 kg) were obtained the CHCl_3_ extract (53.8 g) and MeOH extract (158.3 g). From the leaves of *Davilla elliptica* (1 kg) were obtained the CHCl_3_ extract (14.8 g) and MeOH extract (120.5 g).The flavonoid-rich fractions were obtained after a liquid-liquid extraction step with a portion (10.0 g) of MeOH extract between ethyl acetate and water (1:1, v:v). After the liquid-liquid extraction the both layer were concentrated to yield the organic and aqueous fractions.

### Venom

A pool of lyophilized *Bothrops jararaca* venom was obtained from the Butantan Institute. The venom was maintained at – 20^o^C until use and venom solutions were prepared with PBS. The amount of venom was expressed by protein content. Protein determination was performed according to the method of Bradford [24] using the Bio-Rad Protein Assay Kit. 

### Animals

Swiss male mice weighing 25-30 g were obtained from Central Animal House of UNESP-Botucatu (Botucatu, SP, Brazil). The UNESP Institutional Animal Care and Use Committee approved the employed protocol. The animals were fed a certified Nuvilab CR-a^Ò^ (Nuvital) diet with free access to tap water under standard conditions of 12 h dark-12 h light and temperature (21^o^C ± 1 %).

### Determination of minimum hemorrhagic dose (MHD)

Animals were divided randomly into six groups of 6-7 animals each. The animals received increasing doses of venom (minimum 1.25 µg, maximum 20 µg) injected by an intradermic route into shaved dorsal skin under ether anesthesia. Two hours later, mice were killed by cervical displacement and the diameter of the hemorrhagic area was measured by a Mitutoyo^®^ digital pachymeter [25]. The minimum hemorrhagic dose (MHD) was the lowest dose of venom that induced a hemorrhagic area of 10 mm diameter in 2 h [26]. The method of approximate quantification of hemorrhagic activity by averaging diameter measurements of the hemorrhagic haloes [12] obtained after intradermic injection of venom is specific, rapid and reproducible. This method of quantifying hemorrhagic lesions measured the hemorrhagic halo present on the internal face of dorsal skin of the experimental animals. The quantification consists of reading the two largest perpendicular diameters of the halo, using the average to reveal the size of the hemorrhagic halo (mean diameter). This method could be employed to determine whether the anti-venom substances alter homeostasis or cause blood vessels to rupture. However, in some cases hemorrhagic haloes are irregular. In order to minimize this measurement error, we also used another parameter that consists of the area hemorrhagic obtained by Av Soft Bioview Spectra. The image analysis method enables determination of the hemorrhagic areas of any shape, resulting in readings that are more reliable than those obtained by measuring lesion diameters.

### Neutralization of hemorrhagic effect

For our study of neutralization of *B. jararaca* venom, a fixed amount of venom and extract was used (1:50) so as to assure the interaction between venom and substances present at low concentrations in extracts. When the objective is to detect anti-hemorrhagic compounds in plants, this methodology is more selective than experiments based on injecting venom independent of extract [17].

Venom inhibition assays consisting of solutions containing a fixed amount of venom (1MHD) were mixed with extract or fraction, at 1:50 venom to extract or fraction (w:w) ratio and incubated for 40 minutes at ambient temperature (20-23^o^C). 

The animals in each experiment were divided in groups that received only PBS (to assure the vehicle is innocuous), venom without extract or fraction (positive control), only extract or fraction (to assure the plant is innocuous) and venom plus extract or fraction (to evaluate anti-hemorrhagic potential of plant species). Two hours after the injection, mice were killed by cervical displacement. The neutralization potential of extracts or fractions was evaluated through mean diameter (mm) captured by a Mitutoyo^®^ digital pachymeter while the hemorrhagic area (mm^2^) was obtained by the AvSoft Bioview Spectra^®^ program, comparing the group that received only venom to animals that received intradermic injection of venom plus extract or fraction mixture. All animals that received only PBS (vehicle), extracts or fractions without venom not present hemorrhagic diameter or hemorrhagic lesion (data not shown).

### Statistical analysis

The statistical analysis was based on ANOVA followed by Dunnett’s test. A p value < 0.05 was considered a significant response.
